# Effect of Reducing Nitrogen Fertilization and Adding Organic Fertilizer on Net Photosynthetic Rate, Root Nodules and Yield in Peanut

**DOI:** 10.3390/plants12162902

**Published:** 2023-08-09

**Authors:** Guanchu Zhang, Qiangbo Liu, Zhimeng Zhang, Dunwei Ci, Jialei Zhang, Yang Xu, Qing Guo, Manlin Xu, Kang He

**Affiliations:** 1 Shandong Peanut Research Institute, Qingdao 266100, China; guanchuzhang@126.com (G.Z.); qinhdao@126.com (Z.Z.); cdw_2007@126.com (D.C.); xy52120092661@163.com (Y.X.); jone007@126.com (Q.G.); xumanlin@126.com (M.X.); 2 National Key Laboratory of Wheat Improvement, College of Life Sciences, Shandong Agricultural University, Tai’an 271018, China; liuqiangbo@sdau.edu.cn; 3 Shandong Academy of Agricultural Sciences, Jinan 250100, China; zhanglei840606@126.com

**Keywords:** peanut, nitrogen fertilizer, organic fertilizer, photosynthetic characteristics, root nodules

## Abstract

Long-term excessive application of chemical fertilizers can cause many problems, such as soil degradation and environmental pollution. Therefore, we reduced conventional nitrogen fertilization and added organic fertilizers in some cases to investigate the response of photosynthetic characteristics, root nodules and yield on reduced nitrogen fertilization. Compared to conventional nitrogen fertilization, the 25% and 35% nitrogen reduction treatments reduced the leaf area index, net photosynthetic rate, 100-fruit weight, 100-kernel weight and the yield of peanut, but had no significant effect on the kernel rate. With constant N fertilizer, adding organic fertilization alone increased leaf area index, chlorophyll, net photosynthetic rate and yield of peanut. In compounded treatments of nitrogen and organic fertilizer, the highest yields were achieved in the 25% N reduction with the 3000 kg/hm^−2^ organic fertilizer treatment (T3) and the 4500 kg/hm^−2^ organic fertilizer treatment (T4); furthermore, the net photosynthetic rate, leaf area index, yield and fertilizer contribution were significantly higher in these two treatments than in the conventional fertilizer treatments. Nitrogen fertilizer had significant effects on the quantity and fresh weight of root nodules. Concretely, nitrogen reduction increased the quantity and fresh weight of root nodules of peanut in the early stage of fertility but decreased them in the harvest stage. Nitrogen reduction with an additional organic fertilizer in the late stage of fertility increased the quantity and fresh weight of root nodules of peanut. Considering the property of root nodules was significantly positively correlated with net photosynthetic rate and yield, the arguments above may be the mechanism of the highest yields found in T3 and T4. This work can provide empirical and instructional support for a balanced fertilization strategy in peanut agriculture and high-yielding and efficient cultivation of peanut.

## 1. Introduction

As an important oil crop in China, peanut occupies an important position in national production, and with the development of society, the demand has further increased. However, with the increase in demand, the continuous crop set and pesticide abuse for the simple pursuit of yield have led to increasingly serious hazards. On the other hand, the use of inorganic fertilizers as the main means of increasing yield in agriculture has brought about a number of problems while increasing production. For example, excessive fertilizers can flow into groundwater and pollute water bodies, while an unreasonable fertilizer mix further aggravates fertilizer leaching. At the same time, excessive fertilizers can cause increased soil salinity, which can lead to secondary salinization and seriously threaten soil health [1,2,3]. Currently, our chemical fertilizer utilization rate is less than 40%, and losses vary in different soil environments; for example, nitrogen fertilizer utilization rate is less than 30% in saline soils; therefore, reducing chemical fertilizer use by incorporating organic fertilizers is a new measure to ensure yield and soil health today [4].

Organic fertilizers have elements essential for plant growth and can directly improve crop yield and quality without harming the environment [5,6]. Zhao et al. [7] reported the highest yield of rice under the combined application of chemical fertilizers and organic fertilizers compared to chemical fertilizers alone and attributed this to improved nitrogen utilization efficiency. Moe et al. [8] also reported that the combined use of chemical and organic fertilizers was more beneficial to plant growth for yield enhancement compared to chemical fertilizers alone. However, it has also been reported that the combined use of fertilizer and manure has a negative effect on nitrogen uptake, which may be attributed to differences in crop and growing environment. Therefore, more work is needed to verify whether the combined use has a more effective yield enhancement effect [9].

Rhizobia, as a symbiotic strain of legumes, can convert nitrogen in the air into nitrate nitrogen that can be absorbed by the roots due to its unique nitrogen fixation function [10]. Therefore, fully enhancing the nitrogen fixation capacity of rhizobia has an important role in reducing nitrogen fertilizer use. For example, Wu et al. [11] reported the total N supply sources over the whole growth stage mainly came from root nodules, soil and N fertilizer. The N supply proportion from root nodule, soil and N fertilizer was 5:3:2. Etsami et al. [12] similarly found that the increased nitrogen fixation capacity of rhizobia directly contributed to yield increase.

However, it has also been reported that the reduced use of nitrogen fertilizer did not have a significant effect on rhizobia [13,14]. And the current reports mainly focus on the effect of reduced fertilizer use on crop yield but less on the growth of peanut and the rhizobia response [15]. Therefore, this experiment was conducted to investigate the effect on peanut growth under reduced use of inorganic fertilizers by combining chemical fertilizers with organic fertilizers. On the other hand, the mechanism of combined use on peanut and rhizobia growth was investigated by examining the photosynthetic rate and rhizobia growth.

## 2. Results

### 2.1. Effect of Nitrogen Fertilization with Organic Fertilizer on Photosynthetic Characteristics of Peanut

**Figure 1:**
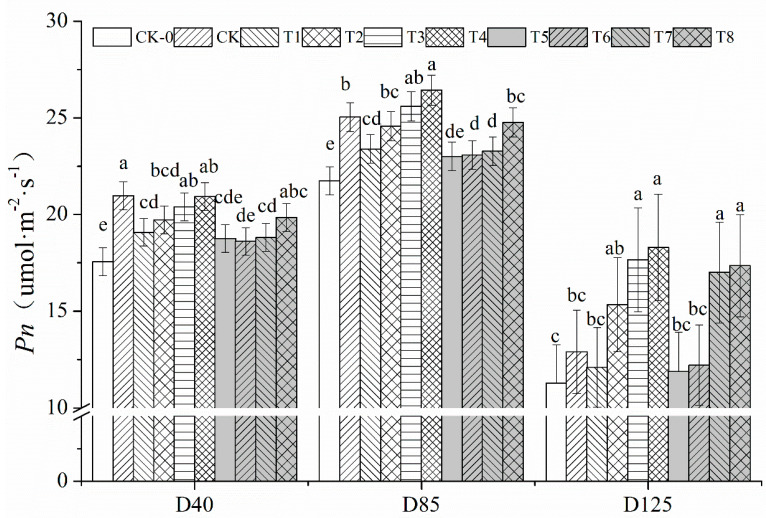
**The *Pn* of leaf in peanut under different treatments.** Different lowercase letters mean significant differences at 0.05 level.

Reduced N fertilizer application reduced the net photosynthetic rate of peanut leaves (Figure 1). At D40, T1, T5 and CK-0 were reduced by 9.02%, 10.55% and 16.28%, respectively, compared with CK; at D85, T1, T5 and CK-0 were reduced by 6.57%, 8.11% and 13.14%, respectively, compared with CK; at D125, T1, T5 and CK-0 were reduced by 6.23%, 7.87% and 12.46%, respectively, with significant differences between the nitrogen reduction treatments and CK at both D40 and D85. Nitrogen reduction with organic fertilizer increased the net photosynthetic rate of peanut leaves, with T2, T3 and T4 increasing 5.05%, 9.43% and 12.99% compared to T1, and T6, T7 and T8 increasing 2.93%, 11.81% and 7.64% compared to T5 at D85, and significant differences water were reached under T3, T4 (compared to T1) and T8 (compared to T5) treatments. The gap was further increased at the late stage of growth (D125), where T3, T4, T7 and T8 all reached significant differences in net photosynthetic rate compared to the control treatment.

**Figure 2:**
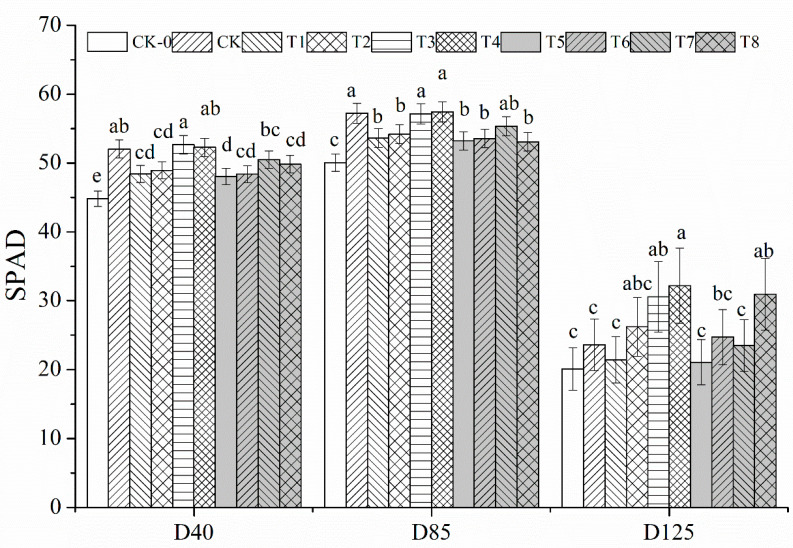
**The SPAD of leaf in peanut under different treatments.** Different lowercase letters mean significant differences at 0.05 level.

Similarly, reduced N fertilizer application reduced peanut leaf SPAD (Figure 2). At D40, T1, T5 and CK-0 were reduced by 6.94%, 7.66% and 13.87%, respectively, compared to CK; at D85, T1, T5 and CK-0 were reduced by 6.25%, 6.98% and 12.51%, respectively, compared to CK; at D125, T1, T5 and CK-0 were reduced by 9.17%, 10.65% and 14.78%, respectively, reaching a significant difference. However, N reduction with organic fertilizer increased peanut leaf SPAD values, with T2, T3 and T4 increasing by 4.77%, 9.14% and 10.74% compared to T1, and T6, T7 and T8 increasing by 17.31%, 11.43% and 46.77% compared to T5 at D125, and significant differences were reached at T4 and T8. Overall, leaf SPAD values were not reduced in T3 and T4 treatments compared to CK at D40, D85 and D125.

**Figure 3:**
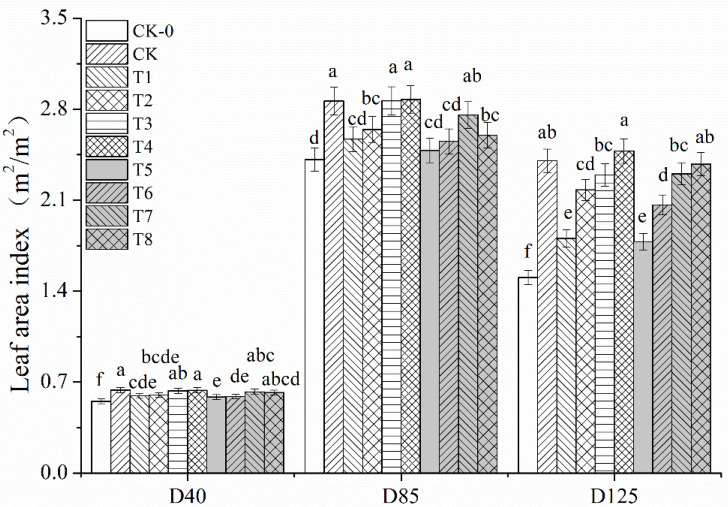
**The LAI of leaf in peanut under different treatments.** Different lowercase letters mean significant differences at 0.05 level.

Reduced nitrogen fertilizer application reduced the leaf area index of peanut (Figure 3). The leaf area index of T1, T5 and CK-0 were lower than CK at D40, D85 and D125, and the difference was significant. Nitrogen reduction with organic fertilizer increased the leaf area index of peanut, and at D125, T2, T3 and T4 decreased 20.67%, 27.06% and 37.17%, respectively, compared with T1, and T6, T7 and T8 decreased 16.04%, 29.50% and 33.66%, respectively, compared with T5, with significant differences. At D40, the leaf area index under T2, T3 and T4 treatments was the same as that under conventional fertilization (CK) were basically the same, interestingly, at D85, T3 and T4 increased by 0.014% and 0.39% compared to CK, but the difference was not significant.

### 2.2. Effect of Nitrogen Reduction with Organic Fertilizer on Peanut Root Nodules

Table 1 shows that nitrogen fertilization had significant regulatory effects on the number of root tumors and fresh weight of root nodules, and nitrogen reduction increased the number of root nodules and fresh weight of peanut root nodules in the early reproductive period, while it decreased the number of root nodules and fresh weight of peanut root nodules in the harvest period, 91.67%, 116.67%; at D85, the number of T1, T5, CK-0 root nodules increased by 32.98%, 62.41%, 125.88%, and the fresh weight of root nodules increased by 33.87%, 62.90%, 125.81% compared with CK; at D125, the number of T1, T5, CK-0 root nodules decreased by 7.69%, 26.77%, 31.80% compared with CK, 31.80%, and 8.0%, 26.01%, 32.04% lower fresh weight of root nodules compared to CK.

In contrast, N reduction with organic fertilizer increased the number of root nodules and fresh weight of peanut roots in the late reproductive stage, and at D125, the number of root nodules increased by 20.73%, 29.29%, 46.15%, 20.85% and 22.78% in T2, T3, T4, T7 and T8 compared with CK; the fresh weight of root nodules increased by 2%, 30%, 48%, 22% and 22% in T2, T3, T4, T7 and T8 compared with CK, T3 and T4 were the highest in both number of root nodules and fresh weight of root nodules.

### 2.3. Effect of Nitrogen Reduction with Organic Fertilizer on Yield Traits of Peanut

As seen in Table 2, nitrogen reduction reduced yield, 100-fruit weight and 100-kernel weight of peanut but had a non-significant effect on yield; T1, T5 and CK-0 yield decreased by 19.26%, 36.49% and 47.86% (2018) and 20.72%, 32.13% and 40.69% (2019), respectively, compared to CK, with differences reaching significant levels; T1, T5 and CK-0 100-fruit weight decreased by 6.80%, 7.39% and 8.11% (2018), respectively, and 3.67%, 3.97% and 4.59% (2019), respectively, compared to CK, and the differences reached significant levels; T1, T5 and CK-0 100-kernel weight decreased by 6.01%, 6.34% and 22.49% (2018), respectively, compared to CK, and 4.16%, 4.29% and 10.43% (2019), and the differences reached a significant level.

In contrast, the application of organic fertilizer with N reduction increased peanut yield, 100-fruit weight and 100-kernel weight, T2, T3 and T4 yield increased by 1.09%, 28.12% and 28.71% (2018),14.85%, 32.65% and 33.45% (2019), respectively, compared to T1, T6, T7 and T8 yield increased by 0%, 1.72%, 11.86% (2018) and 13.58%, 21.13%, 43.45% (2019), respectively. The two-year variation trends of 100-fruit weight and 100-kernel weight were more consistent with the yield. The yield changes of each treatment were T4 > T3 > CK > T2 > T1 > T8 > T7 > T6 > T5 > CK-0 (2018), for T4 > T3 > CK > T8 > T2 > T7 > T1 > T6 > T5 > CK-0 (2019). The maximum yield was shown in T4 and T3 treatments (7216.83~7272.38 kg/hm^−2^), achieving significant differences compared to CK.

### 2.4. Effect of Nitrogen Reduction Combined with Manure on Fertilizer Contribution Rate

**Figure 4:**
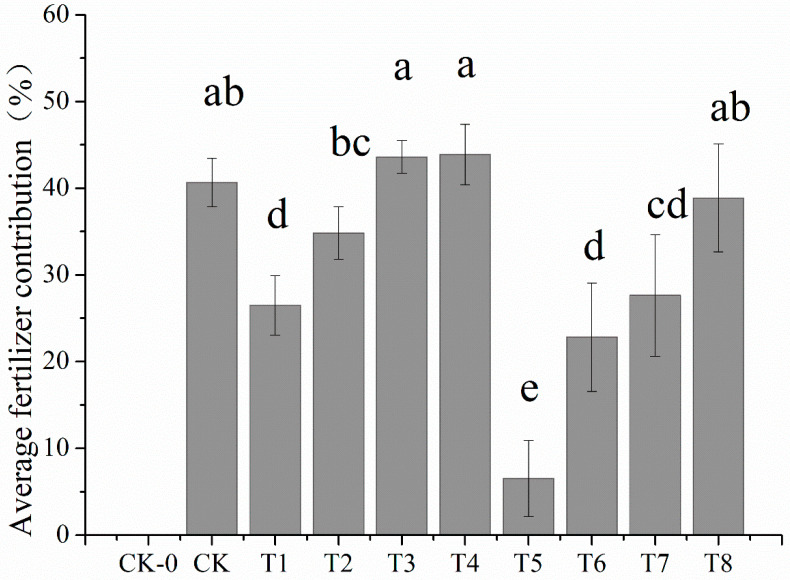
Average fertilizer contribution under different treatments. Different lowercase letters mean significant differences at 0.05 level.

Compared with CK (Figure 4), nitrogen reduction application reduced the average contribution rate of fertilizer, but the combined application of Manure increased the contribution rate of fertilizer. The fertilizer contribution rates among different treatments are T4 > T3 > CK > T2 > T1 > T8 > T7 > T6 > T5 > CK-0. Overall, T4 and T3 are the highest, which are 7.30% and 7.95% higher than CK, respectively.

### 2.5. Linear Analysis of Net Photosynthetic Rate, Root Nodules and Yield

**Figure 5:**
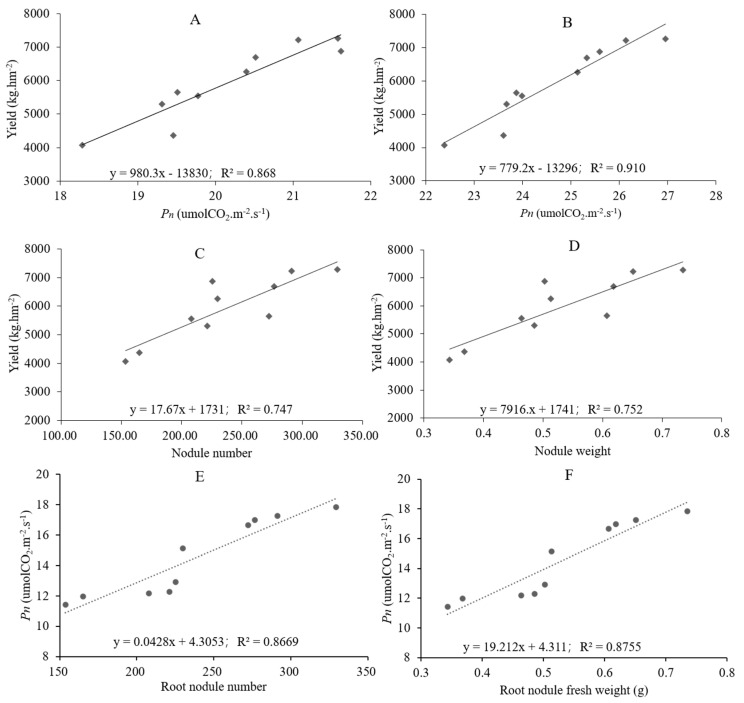
Linear analysis of net photosynthetic rate, root nodules and yield. (**A**,**B**): Linear analysis of yield and *Pn*; (**C**): Linear analysis of yield and nodule number; (**D**): Linear analysis of yield and nodule weight; (**E**): Linear analysis of *Pn* and root nodule number; (**F**): Linear analysis of *Pn* and root nodule weight.

In order to explore the relationship between net photosynthetic rate, root nodules, and yield, we further conducted correlation analysis on the sample properties of all treatment groups. The results showed (Figure 5A,B) that at D40 and D85, the *Pn* was significantly positively correlated with yield (at D125, *Pn* was positively correlated with yield, but the R^2^ value was too small to be listed), indicating a good positive correlation between *Pn* and yield under all different treatments. Furthermore, we investigated the correlation between root nodule number, root nodule fresh weight and yield, and the results showed (Figure 5C,D) that at D125, the number and fresh weight of root nodules were also significantly positively correlated with yield, with R^2^ being 0.747 and 0.752. Respectively, we also conducted a linear analysis on the number of root nodules, fresh weight of root nodules, and *Pn* of peanuts in the later growth stage (D125). The results showed a significant positive correlation (Figure 5E,F). This indicates that the root nodules can continue to affect the net photosynthetic rate of peanuts in the later growth stage, thereby further affecting yield.

## 3. Discussion

### 3.1. Response of Plant Morphology and Photosynthetic Characteristics to Different Fertilization Treatments

Nitrogen is an essential growth element for plant growth and is a component of substances such as proteins, nucleic acids and chlorophyll. The uptake and utilization of nitrogen by plants are influenced by many factors such as species, geology and water and fertilizer supply. Within a certain range of nitrogen fertilizer applications, increased nitrogen fertilization can significantly improve photosynthesis, carbon and nitrogen metabolism, and increase yield [16,17]. However, excessive application of nitrogen fertilizer can lead to aboveground nutrient apparition of peanut, increased leaf area, susceptibility to collapse, greed and late maturity and reduced population quality [18,19]. Other reports showed that the nitrogen application significantly improved the photosynthetic performance of peanuts, especially the photosynthetic rate and respiratory rate of peanut population during the flowering and needle stage [19]. Gohari et al. [20] found that within a certain range of nitrogen application, the pod yield of peanut increased with the increase of nitrogen application, and after exceeding the optimum nitrogen application, the pod yield no longer increased or had a tendency to decrease [21,22]. However, Lombin et al. [23] concluded that nitrogen fertilization had no effect on the yield increase in peanut. The results of this experiment showed that both 25% and 35% N reduction reduced the main stem height, leaf area index, SPAD value and net photosynthetic rate of peanut; in the middle and late stages of fertility, additional organic fertilizer increased the net photosynthetic rate of leaves, and the net photosynthetic rate and SPAD of T3 and T4 treatments were the highest under the synergistic application of N fertilizer and organic fertilizer, which may be explained as follows: N in chemical fertilizer can be released rapidly and thus directly replenish the soil N The nutrients in bio-organic fertilizers need to be transformed by microbial processes before they can be absorbed by plants, and this process is longer than that of chemical fertilizers, therefore, the physiological indexes of peanut decreased rapidly under the rapid nitrogen reduction treatment, while in the middle and late stages, the photosynthetic efficiency and yield of peanut were improved under the treatment of additional organic fertilizers [24,25].

### 3.2. Response of Peanut Root Tumor Number and Root Tumor Fresh Weight to Nitrogen Reduction with Organic Fertilizer Application

As a legume, its nitrogen nutritional characteristics are different from other non-nitrogen fixing crops, and the root system is symbiotic with rhizobia, which can convert atmospheric nitrogen into organic nitrogen fixed in the plant and has a significant effect on nitrogen accumulation and yield of peanut. Tajima et al. [26] found that the nitrogen fixation capacity of rhizobia varies greatly depending on the amount of fertilizer applied and variety type, and nitrogen fertilizer transport can coordinate to promote root system Nitrogen fertilization can coordinate the development of root system and root nodules, and the application of N fertilizer can affect the infestation of rhizobia, the development of root nodules and the nitrogen fixation capacity of root nodules [11,27]. Qiang et al. [28] found differences in the response of nitrogen-efficient rape varieties to different levels of nitrogen supply, and the cumulative nitrogen uptake of nitrogen-efficient varieties was higher than that of nitrogen-inefficient varieties under low nitrogen stress, and similar conclusions were reached in rice [29] and peanut [30]. Yong et al. [31] found significant differences in indicators such as nitrogen fertilizer utilization and nitrogen fertilizer bias productivity among peanut varieties, and the application of moderate amounts of nitrogen fertilizer facilitated the formation and growth of root nodules in peanut roots and improved the nitrogen fixation capacity of root nodules. In contrast, Qiao et al. [32] found that sufficient nitrogen application inhibited nodule primordium development to nodule and nodule growth. Yongmi et al. [27] found that the lower level of nitrogen fertilization in the early stage, the greater number of rhizobia-infested nodules and root tumor weight, and also explored that the nitrogen fixation and supply capacity of peanut root tumors determines the total nitrogen accumulation of the plant. The results of this experiment indicate that different nitrogen fertilizer treatments have a significant impact on root nodules. The number and fresh weight of root nodules were significantly increased by nitrogen reduction combined with organic fertilizer, among which T3 and T4 treatments reached the maximum value (Table 1). Further, through linear analysis, the number and fresh weight of root nodules were significantly positively correlated with *Pn*, while *Pn* was also positively correlated with yield, indicating that the increase of root nodules was also one of the reasons for the highest yield under T3 and T4 treatments.

### 3.3. Effect of Nitrogen Reduction and Organic Fertilizer Application on Peanut Yield and Fertilizer Contribution Rate

Crop yield is controlled by a combination of crop varieties, cultivation conditions and soil environmental factors. The improvement of soil nutrient content directly contributes to the increase in crop yield. Organic fertilizer, as an effective soil additive, has a long-term effect on improving soil properties and fertility levels. In particular, it increases soil organic carbon, which is an important indicator for evaluating potential soil productivity and soil quality and is closely related to crop growth [33]. The addition of organic fertilizers also promotes the increase of soil microbial diversity and changes in enzyme activity, accelerating the mineralization and utilization of nutrients. At the same time, the combined use of organic fertilizers with inorganic fertilizers further improves soil properties while directly increasing soil fertility levels while promoting long-term fertilizer release, thus making it more favorable as yield increases. On the other hand, the combined use reduced fertilizer leaching while increasing the utilization of phosphorus and potassium fertilizers by the crop and significantly increased the crop yield compared to the single application of fertilizers [2,3].

## 4. Method and Material

### 4.1. Growth Conditions

The experiment was conducted from 2018 to 2019 in Yinan County (E: 118.471, N: 35.5513), Linyi City, Shandong Province. This area has a warm temperate monsoon climate, with average annual temperatures of 8–18 °C. The soil type of the test field is cinnamon soil. The physical and chemical properties of the base soil of the cultivated layer were tested (Table 3).

### 4.2. Materials

We used the local main peanut variety named Long Hua 128. 120 kg/hm^−2^ of phosphorus fertilizer was applied, 90 kg/hm^−2^ of potassium fertilizer was applied, the fertilizer of phosphorus and potassium was potassium dihydrogen phosphate (51% P_2_O_5_, 33.8% K_2_O), potassium fertilizer was supplemented with potassium sulfate (about 51% K_2_O), nitrogen (N) fertilizer was urea (46% N), and the test organic fertilizer was commercial organic fertilizer (organic calcium ≥ 2%, organic matter ≥ 50%, humic acid ≥ 20%).

### 4.3. Experiment Design

We set 10 treatments in total: without N fertilizer (CK-0), conventional N fertilizer (120 kg/hm^−2^; CK), 25% reduction of N compared to conventional fertilizer (T1), 25% reduction of N with organic fertilizer (organic matter content ≥ 30%, humic acid content ≥ 15%, pH 5.5) added in a gradient (1500 kg/hm^−2^, 3000 kg/hm^−2^ and 4500 kg/hm^−2^; T2 to T4, respectively), 35% reduction of N compared to conventional fertilizer (T5), and 35% reduction of N with additional organic fertilizer in a same gradient (T6 to T8). The fertilizer was mixed thoroughly before land preparation and applied as a basal fertilizer to the corresponding plots with an area of 33.3 m^2^ in a randomized group arrangement with three replicates. Harvested on 20 September 2018 and 2019, each plot has calculated the yields separately.

### 4.4. Methods

#### 4.4.1. Measurement of Photosynthetic Rate and SPAD Values

The net photosynthetic rate (*Pn*) of functional leaves was sampled on days 40, 85 and 125 after sowing using a CIRAS-3 portable photosynthesis system (PP Systems, Amesbury, MA, USA). Measurements were made from 9:00 to 11:00 in clear weather. The leaf blades were measured in the upper middle part of the leaves and avoided the leaf veins, with five replicates of each treatment. SPAD values were measured by SPAD chlorophyll meter (SPAD-502 Chlorophyll Meter Model SPAD-502), with 5 leaves and 3 replicates for each treatment.

#### 4.4.2. Root Nodules Determination

Samples were taken on day 40, day 85 and day 125 after sowing; three randomly selected holes of peanut were taken from 0 to 40 cm soil layer root system, cleaned and then the root tumor was removed with a medical scalpel, the number of root tumors was counted, and the fresh weight of root nodules was weighed with an electronic balance, six replicates.

#### 4.4.3. Yield and Yield Components

The remaining plants were harvested uniformly at harvest time. The rest pods were air-dried. Pods with economic values were randomly selected to calculate the 100-fruit weights, the 100-kernel weights and the kernel rates %.

#### 4.4.4. Data Processing

We analyzed data using SPSS 19.0 data statistical software and used Origin 8.5 for graphing.

## 5. Conclusions

The highest yield was obtained with a 25% N reduction with 4500 kg/hm^−2^ organic fertilizer treatment, and the best benefit was obtained with a 25% N reduction with 3000 kg/hm^−2^ organic fertilizer treatment. Both 25% and 35% N reduction reduced yield compared to conventional fertilizer application. At the same level of N application, additional application of organic fertilizer increased peanut yield. The highest yield in the treatments of 25% N reduction with 3000 kg/hm^−2^ organic fertilizer and 25% N reduction with 4500 kg/hm^−2^ organic fertilizer could be attributed to the high number of root nodules and fresh weight of root nodules maintained by the root system in the late reproductive period, the higher nitrogen fixation efficiency to prevent early plant failure, and the maintenance of high net photosynthetic rate, SPAD and leaf area index to ensure high yield.

## Figures and Tables

**Table 1:** Effect of different treatments on nodule number and fresh weight in peanut.

	D40		D85		D125	
Item	NN	NFW	NN	NFW	NN	NFW
CK-0	154.33 ± 14.98 a	0.26 ± 0.0085 a	360.67 ± 43.29 a	1.40 ± 0.1699 a	153.67 ± 69.37 c	0.34 ± 0.1554 d
CK	73.00 ± 12.29 e	0.12 ± 0.0205 f	159.67 ± 35.57 f	0.62 ± 0.1097 e	225.33 ± 61.52 abc	0.5 ± 0.1369 abcd
T1	112.67 ± 10.69 cd	0.19 ± 0.0083 cd	212.33 ± 10.5 e	0.83 ± 0.0409 d	208.00 ± 48.51 bc	0.46 ± 0.1084 bcd
T2	106.67 ± 9.07 cd	0.18 ± 0.0152 d	237 ± 20.66 de	0.92 ± 0.0803 cd	230.00 ± 58.03 abc	0.51 ± 0.1286 abcd
T3	99.33 ± 4.93 d	0.17 ± 0.0082 e	251 ± 24.88 de	0.98 ± 0.0968 cd	291.33 ± 76.17 ab	0.65 ± 0.1717 ab
T4	118.33 ± 8.96 bc	0.20 ± 0.0152 c	240.33 ± 4.51 de	0.93 ± 0.0176 cd	329.33 ± 81.30 a	0.74 ± 0.1814 a
T5	139.33 ± 7.37 ab	0.23 ± 0.0042 ab	259.33 ± 14.29 d	1.01 ± 0.0056 cd	165.00 ± 41.33 c	0.37 ± 0.092 cd
T6	146.67 ± 13.87 a	0.25 ± 0.0209 a	277.33 ± 37.5 cd	1.08 ± 0.1459 bc	221.33 ± 61.52 abc	0.49 ± 0.1291 bcd
T7	116.67 ± 6.66 cd	0.20 ± 0.011 cd	313.67 ± 17.39 bc	1.22 ± 0.0674 b	272.33 ± 75.43 abc	0.61 ± 0.1649 abc
T8	124.00 ± 16 bc	0.21 ± 0.0267 bc	330.00 ± 31.76 ab	1.28 ± 0.1239 ab	276.67 ± 72.67 abc	0.62 ± 0.1624 abc
Mean	119.1	0.1989	264.13	1.03	237.3	0.5386
SD	23.93	0.0399	59.05	0.23	55.48	0.1243
CV/%	20.09	20.07	22.35	22.41	23.38	23.51

Different lowercase letters mean significant differences at 0.05 level. NN: nodule number. NFW: nodule fresh weight.

**Table 2:** Effect of different treatments on yield characters in peanut.

Year	Treatment	100-Pod Weight/g	100-Seed Weight/g	Kernel Rate/%	Yield/kg·hm^−2^
	CK-0	163.48 ± 3.81 c	52.98 ± 1.37 c	0.65 ± 0.01 b	3637.32 ± 59.62 d
	CK	177.91 ± 3.97 a	68.36 ± 1.59 a	0.77 ± 0.01 a	6976.08 ± 110.28 a
	T1	165.81 ± 2.92 b	64.25 ± 1.56 b	0.78 ± 0.02 a	5632.66 ± 98.26 b
	T2	164.97 ± 2.91 b	64.35 ± 1.52 b	0.78 ± 0.02 a	5694.12 ± 98.26 b
2018	T3	165.38 ± 2.54 b	65.71 ± 1.61 b	0.79 ± 0.01 a	7216.83 ± 185.35 a
	T4	166.32 ± 2.57 b	66.26 ± 1.76 b	0.80 ± 0.01 a	7249.25 ± 176.34 a
	T5	164.76 ± 3.28 b	64.02 ± 1.85 b	0.77 ± 0.02 a	4430.25 ± 86.68 c
	T6	165.15 ± 3.57 b	64.08 ± 1.82 b	0.78 ± 0.02 a	4430.25 ± 157.36 c
	T7	166.67 ± 3.29 b	65.26 ± 2.54 b	0.78 ± 0.01 a	4506.33 ± 128.31 c
	T8	167.24 ± 4.05 b	65.95 ± 2.60 b	0.79 ± 0.01 a	4955.75 ± 114.35 c
	CK-0	162.05 ± 3.51 d	55.11 ± 1.11 c	0.67 ± 0.005 d	4076.3 ± 205.01 f
	CK	169.85 ± 3.55 ab	61.53 ± 1.36 a	0.72 ± 0.004 bc	6873.89 ± 345.95 ab
	T1	164.47 ± 2.87 bcd	58.97 ± 1.47 b	0.71 ± 0.005 c	5449.49 ± 279.23 cde
	T2	168.63 ± 3.76 abc	61.05 ± 1.56 ab	0.72 ± 0.004 bc	6258.98 ± 314.97 bcd
2019	T3	171.24 ± 3.74 a	63.02 ± 1.42 a	0.72 ± 0.006 ab	7229.25 ± 229.78 a
	T4	172.24 ± 3.61 a	62.46 ± 1.55 a	0.72 ± 0.004 bc	7272.38 ± 314.08 bc
	T5	163.11 ± 3.42 cd	58.89 ± 1.39 b	0.72 ± 0.005 bc	4665.03 ± 219.57 ef
	T6	166.89 ± 2.91 abcd	60.37 ± 1.31 ab	0.72 ± 0.004 bc	5298.74 ± 397.81 de
	T7	169.64 ± 2.98 ab	62.38 ± 1.39 a	0.73 ± 0.004 a	5650.71 ± 289.79 bcd
	T8	170.6 ± 2.83 a	62.76 ± 1.64 a	0.73 ± 0.008 a	6692.33 ± 372.54 cde

Different lowercase letters mean significant differences at 0.05 level.

**Table 3:** Soil chemical and physical properties.

Year	SOM	AN	OP	AK	PH
	g·kg^−1^	mg·kg^−1^	mg·kg^−1^	mg·kg^−1^	
2018	12.5	91.84	28.56	95.39	6.0
2019	13.1	92.37	27.75	95.98	6.3

## Data Availability

Not applicable.
